# Supplementary material for: In situ assembly of bioresorbable organic bioelectronics in the brain
Source: Nat Commun. 2023 Jul 24;14:4453. doi: 10.1038/s41467-023-40175-3 (PMC10366153; doi:10.1038/s41467-023-40175-3)
Supplement: Supplementary file 1 — Supplementary Information [file 41467_2023_40175_MOESM1_ESM.pdf]

Supplementary Materials for

In Situ Assembly of Bioresorbable Organic Bioelectronics in the Brain

Martin Hjort[1], Abdelrazek H. Mousa[2], David Bliman[2], Muhammad Anwar Shameem[2], Karin Hellman[1], Amit Singh Yadav[1], Peter Ekström[1], Fredrik Ek[1], and Roger Olsson[1,2]

[1]Chemical Biology and Therapeutics, Department of Experimental Medical Science, Lund University, SE-221 84 Lund, Sweden,

[2]Department of Chemistry and Molecular Biology, University of Gothenburg, SE-405 30 Gothenburg, Sweden.

Corresponding author. Email: roger.olsson@med.lu.se

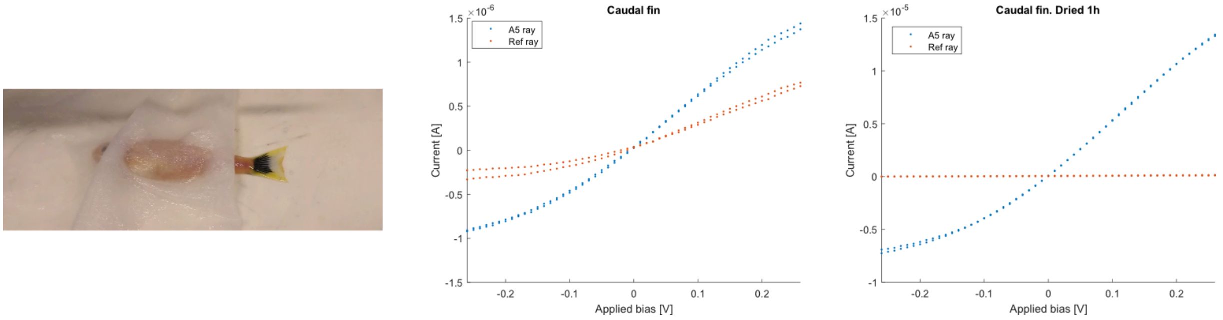

Figure S1. A5 injection into zebrafish caudal fin. Current–voltage measurements of **A5** injected in between caudal fin rays immediately after injection and in a dried fin, respectively. The experiment was repeated 2 times.

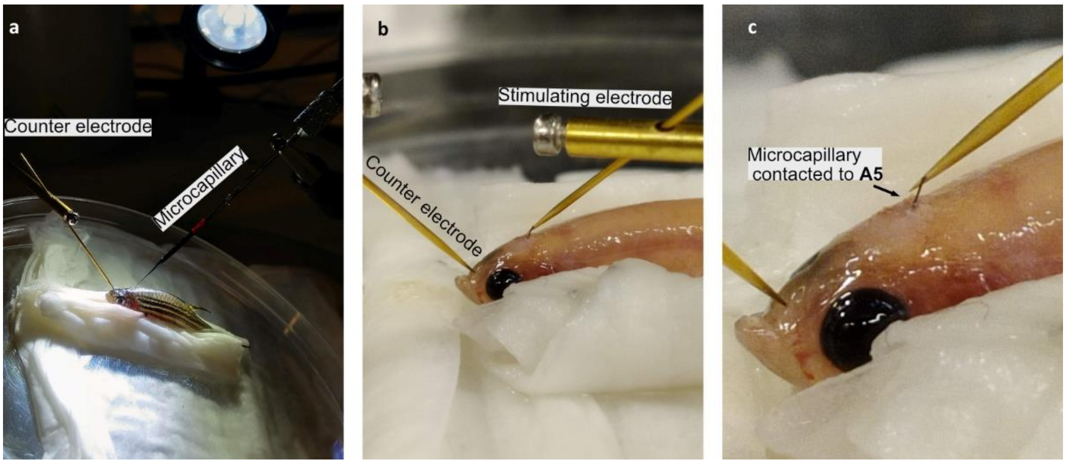

Figure S2. Electrofunctionalization in brains. (**a**) Anesthetized zebrafish injected with **A5** and **ETE-S** using an iridium-coated microcapillary. Following injection, the microcapillary is connected to an external power source and used to electrofunctionalize the **ETE-S**. (**b,c**) Zebrafish after electropolymerization with a broken-off microcapillary still inside the brain and connected to the **A5–ETE-S** electrode. A gold microelectrode can be used to contact the protruding microcapillary.

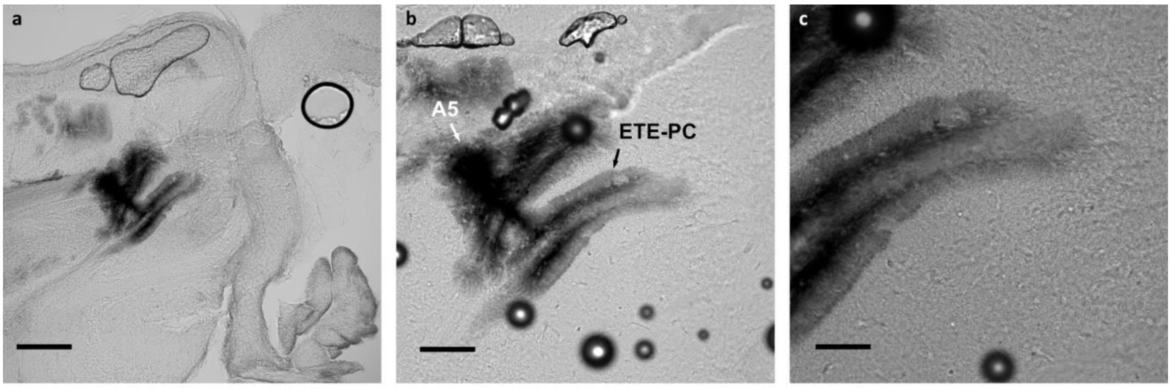

Figure S3. Electrofunctionalization A5–ETE-PC in zebrafish brain slices. Brain sections depicting **A5–ETE-PC** 1 d after electrofunctionalization. The experiment was repeated 3 times. Scalebars denote 200 µm (**a**), 100 µm (**b**), and 50 µm (**c**).

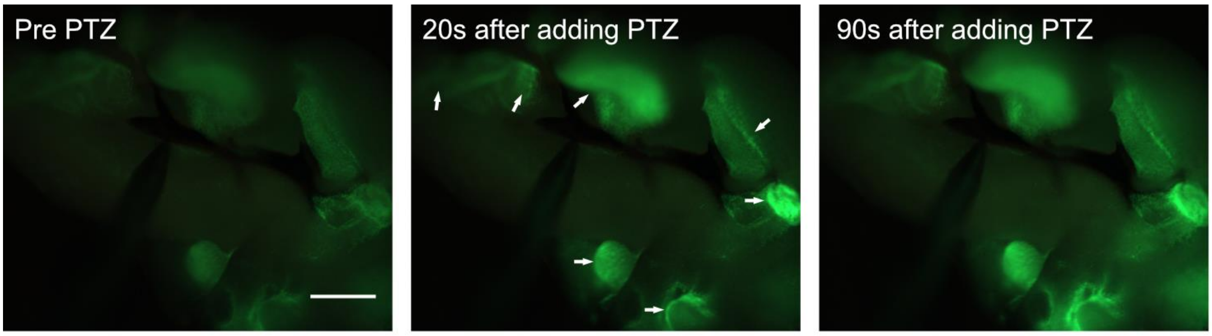

Figure S4. PTZ stimulation in brain slices: After repeated electrical stimulation in the brain slice depicted in Fig. 5, we added **pentylenetetrazole** (**PTZ**) to the imaging medium at a final concentration of about 10mM. **PTZ** is a GABA-A receptor antagonist stimulating unspecific neural activity by blocking GABAergic inhibitory signaling. The experiment was repeated 2 times. Scalebar denotes 500 µm.

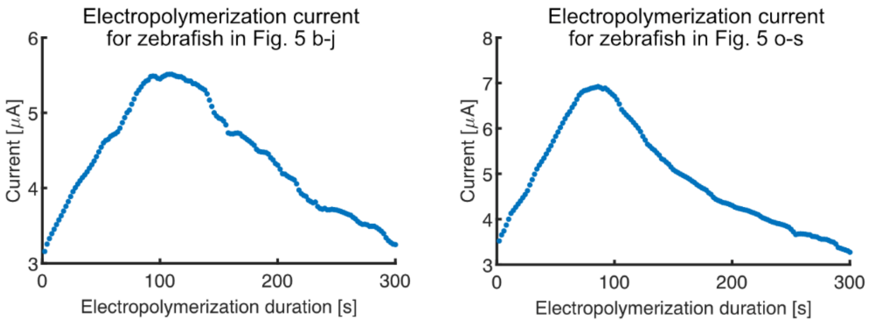

Figure S5. Current flow during *in vivo* electrofunctionalization of A5+ETE-S. 1.5 V was applied between the microcapillary and counter electrode during functionalization.

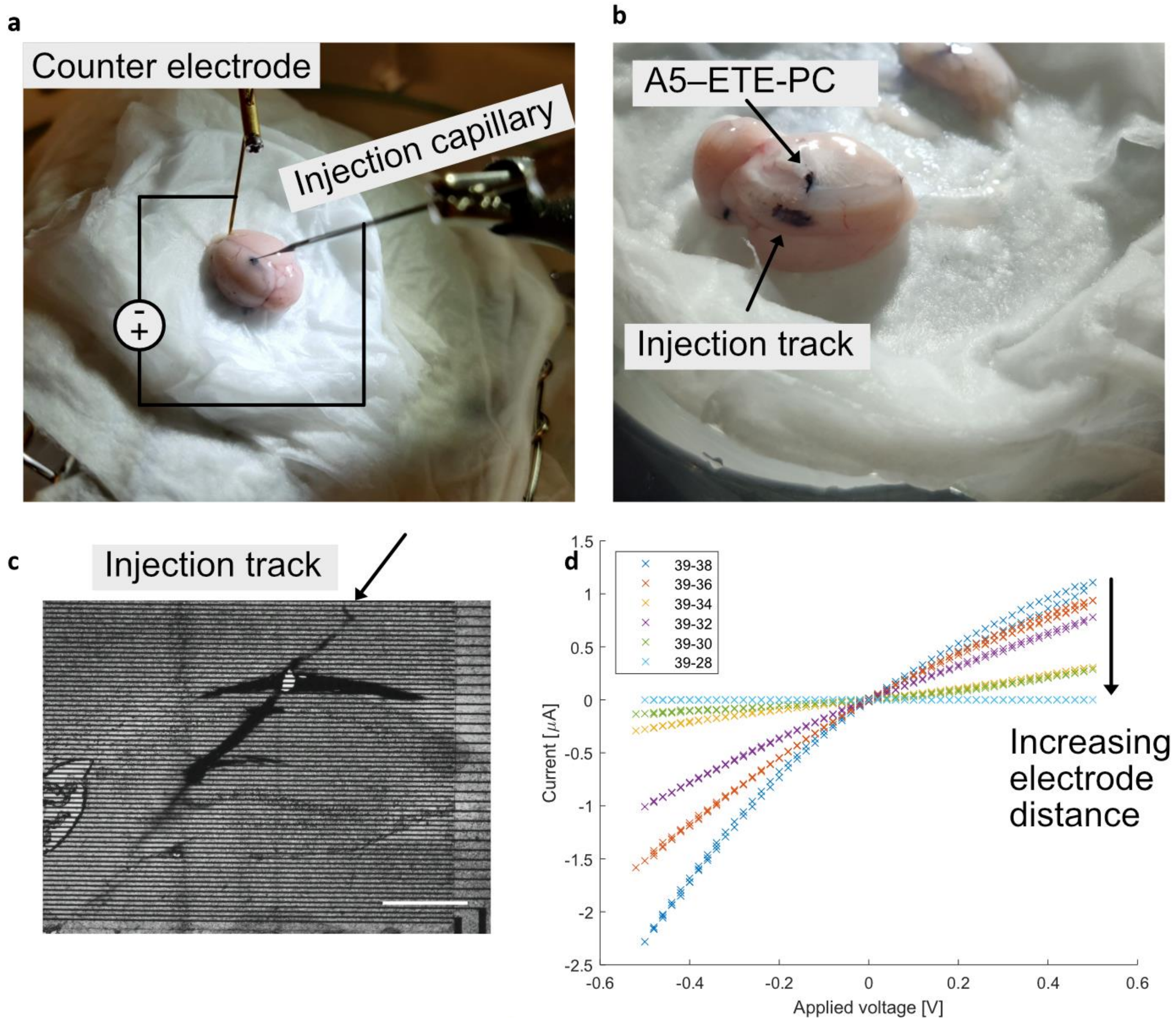

Figure S6. ***Ex vivo*** **electrofunctionalization of A5+ETE-PC in a mouse brain**. (**a**) Setup for injection and subsequent electropolymerization. An Ir-coated microinjection capillary (same as was used for the zebrafish brain injections) was used to inject **ETE-PC+A5** and as the anode for electrofunctionalization at 1.5 V. (**b**) After electrofunctionalization, a dark polymer gel electrode was found along the injection track, similar to what was observed in the Zebrafish studies. (**c**) Sectioned PFA-fixed mouse brain put onto interdigitated gold electrodes. The dark **A5–ETE-PC** track extends into the tissue in a similar fashion to the zebrafish brain. (**d**) Current–voltage characteristics when mapping the interdigitated electrodes in (**c**). High currents, which decrease with increased electrode distance were observed. The experiment was repeated 2 times. Scalebar in (**c**) denotes 500 µm.

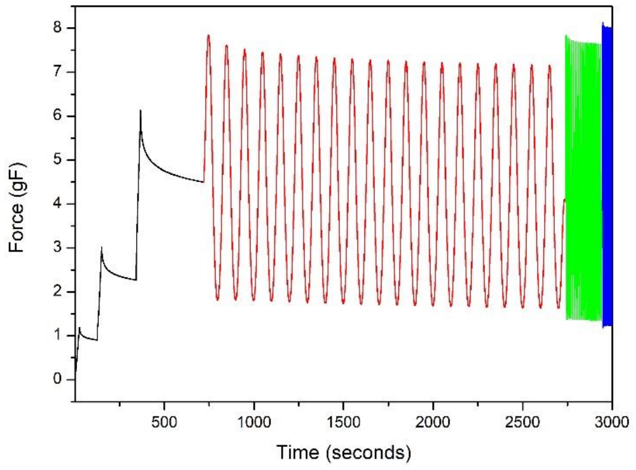

Figure S7. Mechanical testing of electropolymerized A5–ETE-PC. Indentation measurement setup. In black, the 3 stress-relaxations (indentation step followed by relaxation). In red is the dynamic test at 0.1 Hz, green at 1 Hz and blue at 4 Hz.

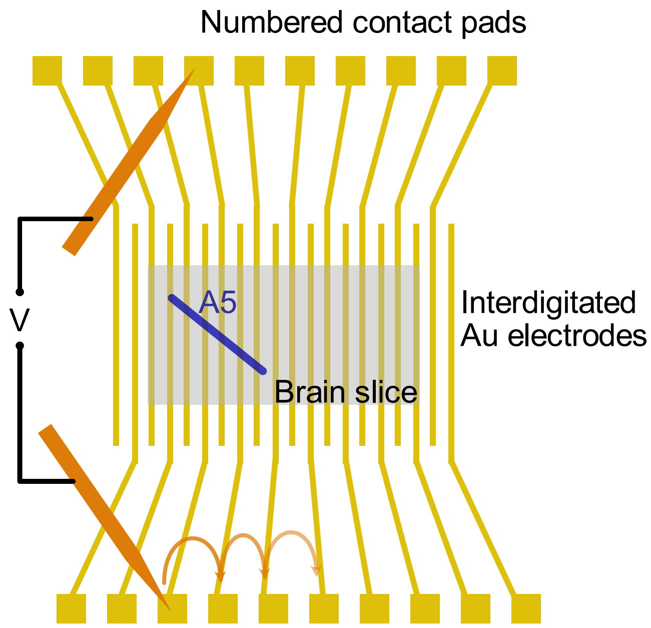

Figure S8. Schematic description of the electrical measurements. Movable external electrodes (orange) are connected to numbered contact pads outside of the brain slice area. The brain slice (light grey) with the injected polymer (dark blue) is placed on the interdigitate Au electrodes. An external voltage is supplied through the external electrodes while the resulting current is registered. The voltage is ramped between -0.5 V and +0.5 V in a cyclic fashion. Once mapped, the external electrodes are moved to different contact pads to map other regions (and the distance dependence) of the injected polymer.

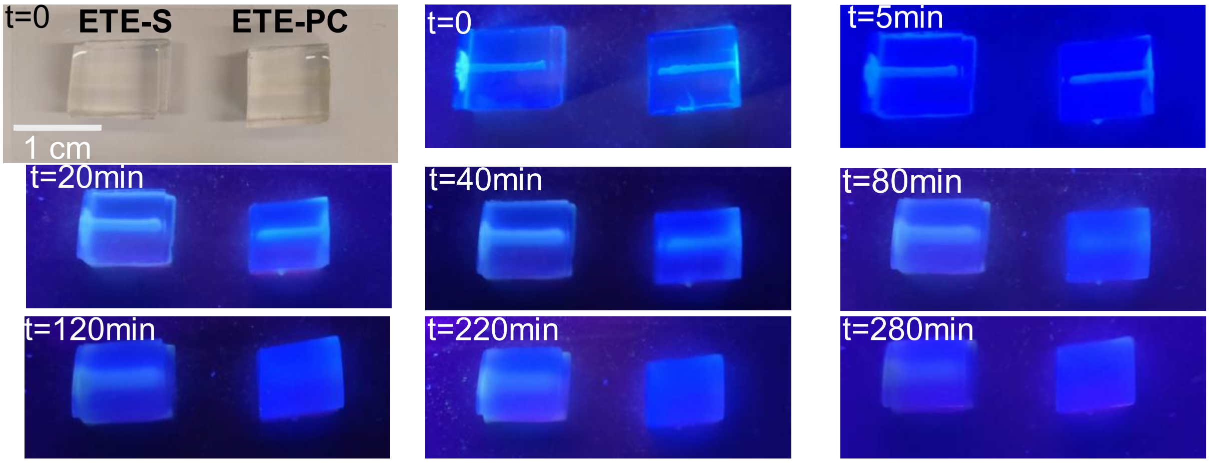

Figure S9. Diffusion of ETE-S and ETE-PC in brain mimicking agarose. 3.5 µl **ETE-R** was injected into 0.6% Ringer–agarose using a Hamilton syringe and left to diffuse. The **ETE-R** can be traced by its fluorescence (excited at 365nm). After 80 min, the **ETE-PC** diffused to the point where it was homogenously distributed in the agarose block. **ETE-S** presented slower diffusion. Images were acquired using a cell phone camera.

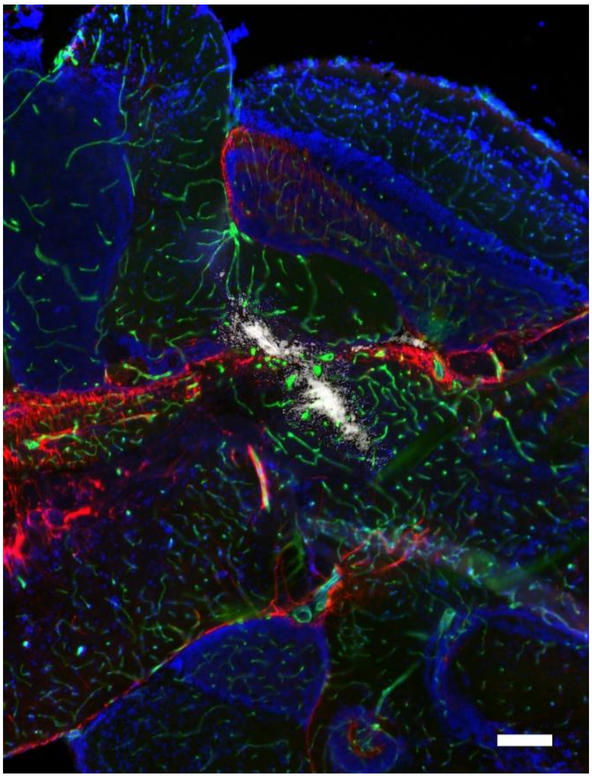

Figure S10. Fluorescence microscopy image of immunostained zebrafish brain section. Brain injected with **A5** (40 mg mL^{-1}) **+ ETE-PC** (40 mg mL^{-1}) and electropolymerized at 1.5V for 5min. Radial glia (anti-GFAP) in red, blood vessels (Fli-GFP) in green, cell nuclei (DAPI) in blue, and polymer-electrode in white. The experiment was repeated 2 times. Scalebar denotes 100 µm.

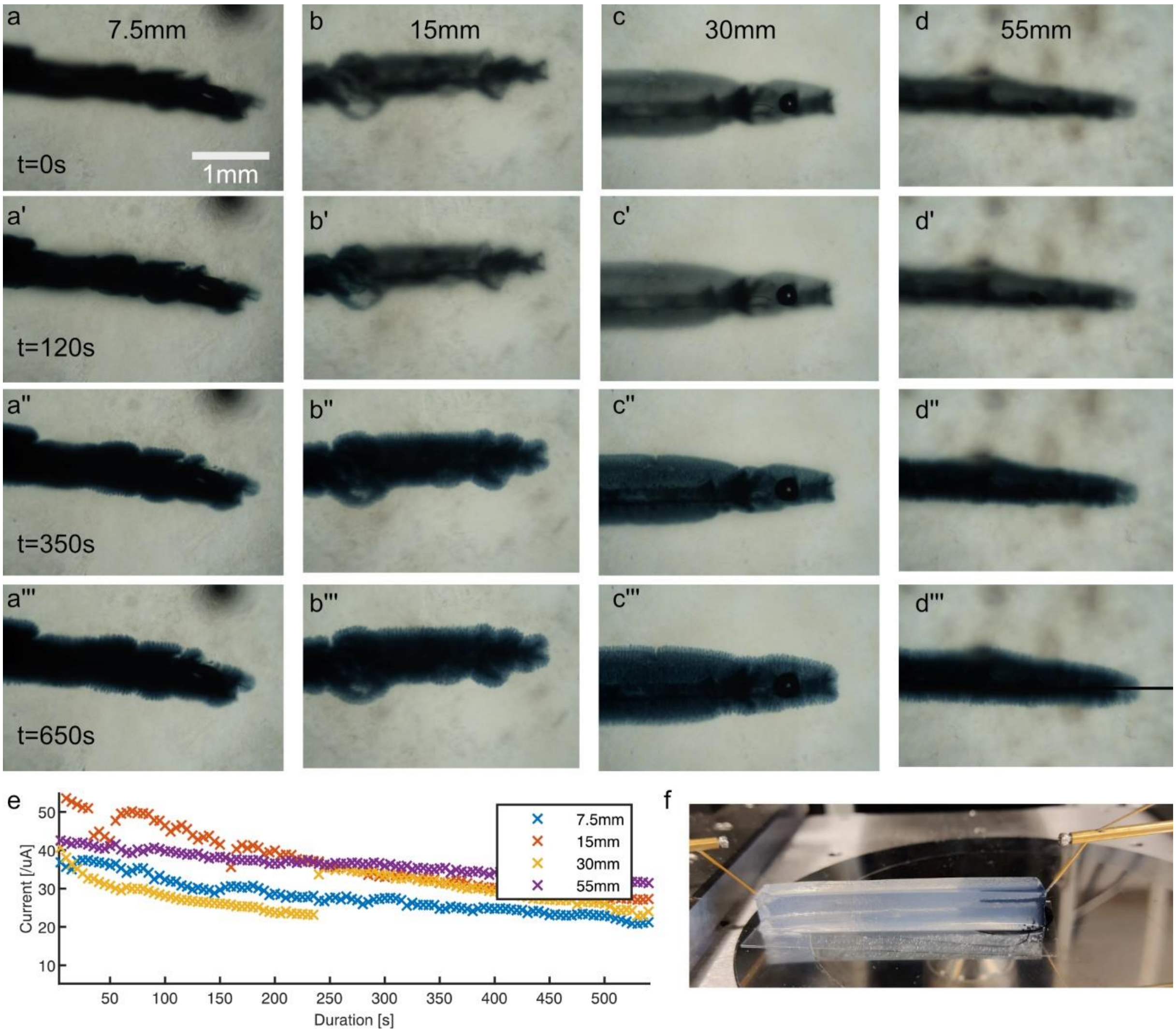

Figure S11. A5+ETE-S electropolymerization at varying electrode distances. 2.5 µl **A5+ETE-S** (20:40 mg mL^{-1}) was injected into 0.6% Ringer agarose using a Hamilton syringe. After injection, the counter electrode (Au-coated W) was placed in the agarose (outside of the **A5+ETE-S**) at varying distances from the electrode connected to the polymer. The **ETE-S** was electrofunctionalized onto the **A5** using a bias voltage of 1.5 V. The polymer electrode was imaged at 0s, 120s, 350s, and 650s. (**a–d**) Brightfield microscopy images depicting **ETE-S** dendrites forming on **A5**. The duration of the applied bias and the electrode distances are indicated in the figure. The electrode distance was not seen to influence the electrofunctionalization process with respect to time, dendrite extension, or current. (**e**) shows the current between the two electrodes during the electrofunctionalization process. (**f**) Photograph of the setup. Scalebar in (**a**) applies to all microscopy images. The experiment was repeated 2 times.

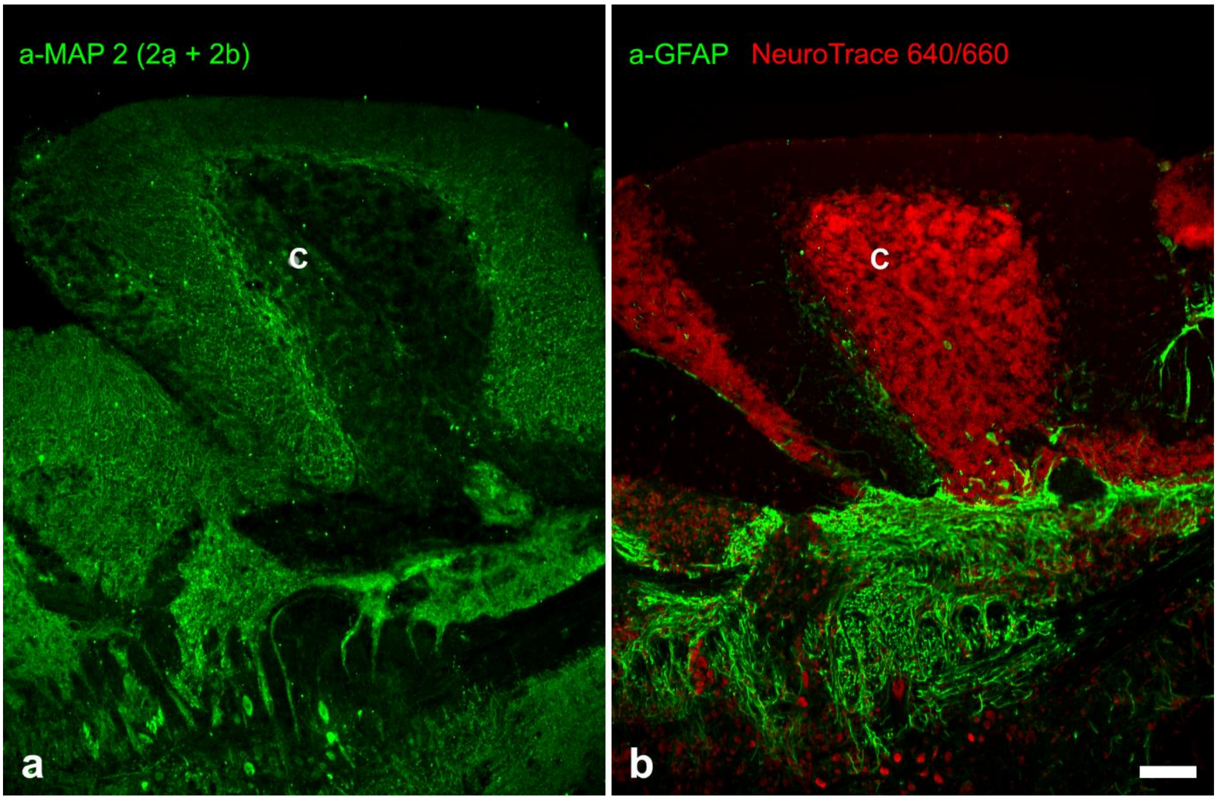

Figure S12. Long-term *in vivo* study, immunofluorescent staining. 11 days after microinjection and electropolymerization of **A5 + ETE-S,** there was no trace of the polymer or injection track in the brain. Immunofluorescence using antibodies against (**a**) microtubule-associated protein (MAP 2 (2a + 2b), a neuronal marker, and (**b**) the glial marker glial fibrillary acidic protein (GFAP) in combination with the fluorescent Nissl stain NeuroTrace 640/60, supplemented with the nuclear stain DAPI (not shown), reveals normal brain histology with no signs of hemorrhage or gliosis around the injection site. (**a**,**b**) show two consecutive 16 µm thick serial sections. C, cerebellum. n=8 fish. Scale bar 100 µm. The experiment was repeated 2 times.
