# Supplementary material for: In situ assembly of bioresorbable organic bioelectronics in the brain
Source: Nat Commun. 2023 Jul 24;14:4453. doi: 10.1038/s41467-023-40175-3 (PMC10366153; doi:10.1038/s41467-023-40175-3)
Supplement: Supplementary file 3 — Description of Additional Supplementary Files Document [file 41467_2023_40175_MOESM3_ESM.pdf]

<u>Description of additional supplementary files document</u>

Movie S1.mp4. Stimulation in brain slices when the A5 is contacted by an external 10 µm tungsten microelectrode. Each stimulation pulse was applied for 5 s and are indicated by a lightning symbol in the movies. 1 frame per second was acquired and the movies are shown at 10x speed. Voltages used for the different stimulation pulses: 11 V, 9 V, and 9 V. The scale bar denotes 500 µm.

Movie S2.mp4. Stimulation is similar to the above, albeit in a different brain slice coming from another fish. Voltages used for the different stimulation pulses: 9 V, 9 V, and 9 V. 1 frame per second, shown at 10x speed. The scale bar denotes 500 µm.

Movie S3.mp4. Spontaneous firing in the regions which were previously stimulated in Movie_S1. The spontaneous firing is stochastic in nature. No stimulation pulses were applied in this movie. 1 frame per second, shown at 10x speed. The scale bar denotes 500 µm.

Movie S4.mp4. Stimulation in brain slices when the external 10 µm tungsten microelectrode contacted the microcapillary outside the brain slice. The capillary was, in turn, contacting the conductive polymer in the slice. Each stimulation pulse was applied for 5 seconds and is indicated by lighting in the movies. 1 frame per second was acquired, and the movies are shown at 10x speed. Voltages used for the different stimulation pulses: 11 V, 9 V, and 9 V. The scale bar denotes 500 µm.

Movie S5.mp4. Fish swimming around with the capillary still inside the skull. The movie was acquired one day after ETE-PC electropolymerization. Real-time movie.

Movie S6.mp4. Electropolymerization of A5–ETE-S in an agarose gel. 1.5 V applied bias. The total movie time was 100 min. The scale bar denotes 1 mm.

Movie S7.mp4. 3D rendered visualization of the light sheet microscopy presented in Fig. 5. Adult zebrafish brain 4 mm.
